# Oral Microbial Signature of Rheumatoid Arthritis in Female Patients

**DOI:** 10.3390/jcm12113694

**Published:** 2023-05-26

**Authors:** Samat Kozhakhmetov, Dmitriy Babenko, Argul Issilbayeva, Madiyar Nurgaziyev, Saniya Kozhakhmetova, Assel Meiramova, Zhanar Akhmetova, Jeanette Kunz, Bayan Ainabekova, Francesco Marotta, Almagul Kushugulova

**Affiliations:** 1 Laboratory of Microbiome, Center for Life Sciences, National Laboratory Astana, Nazarbayev University, Astana Z05H0P9, Kazakhstan; 2 Innovative Center ArtScience, Astana Z00T3X6, Kazakhstan; 3 Department of Internal Medicine with the Course of Gastroenterology, Endocrinology and Pulmonology, NJSC Astana Medical University, Astana 010000, Kazakhstan; 4 National Center for Biotechnology, Astana Z05K8D5, Kazakhstan; 5 Department of Medicine, Nazarbayev University School of Medicine, Astana Z05H0P9, Kazakhstan; 6 ReGenera R&D International for Aging Intervention, 20144 Milan, Italy

**Keywords:** oral microbiome, rheumatoid arthritis, Kazakh female, case-sectional study, KEGG modules

## Abstract

This study aimed to identify the oral microbial signature of Kazakh female rheumatoid arthritis (RA) patients. A total of 75 female patients who met the American College of Rheumatology 2010 classification criteria for RA and 114 healthy volunteers were included in the study. Amplicons of the 16S rRNA gene were sequenced to analyze the microbial composition. We identified significant differences in bacterial diversity and abundance between the RA and control groups, as measured by Shannon (*p* value = 0.0205) and Simpson (*p* value = 0.00152) indices. The oral samples from RA patients had higher bacterial diversity than those from non-RA volunteers. The RA samples had a higher relative abundance of *Prevotellaceae* and *Leptotrichiaceae*, but a lower content of butyrate and propionate-producing bacteria compared to the control group. The samples from patients in remission had a higher abundance of Treponema sp. and *Absconditabacteriales* (SR1), whereas those with low disease activity had higher levels of *Porphyromonas* and those with high RA activity had higher levels of Staphylococcus. A positive correlation was found between the taxa Prevotella_9 and serum levels of antibodies to cyclic citrullinated peptide (ACPA) and rheumatoid factor (RF). The predicted functional pattern of the ACPA+/RF− and ACPA+/RF+ seropositive groups was characterized by increased ascorbate metabolism, degradation of glycosaminoglycans, and reduced biodegradation of xenobiotics. These findings suggest that the functional pattern of the microflora should be considered when selecting a therapeutic strategy for RA in order to provide a personalized approach.

## 1. Introduction

Rheumatoid arthritis is a common autoimmune disease that affects joints and is characterized by the presence of circulating autoantibodies, increased levels of proinflammatory cytokines and chemokines, and a shift in rheumatoid factor [1]. The exact cause of RA is not known, but it is believed to be triggered by various factors such as infections, smoking, and physical or emotional stress. Another trigger of RA is the perturbation of the oral cavity microbial flora. There is evidence to suggest that changes in the microbiome of the oral cavity may be linked to the development of rheumatoid arthritis (RA). In a study conducted by Tong et al., a decrease in the number of *Defluviitaleaceae_UCG-011* and *Neisseria oralis* species was observed in Chinese patients with RA, while an increase in *Prevotella_6* was also seen [2]. Similarly, Kroese et al. found a higher relative abundance of *Prevotella* and *Veillonella* in the saliva of patients with early RA, suggesting a potential connection between the oral microbiome and RA onset [3].

However, the data on microbiome markers for RA are conflicting, with some studies reporting that *Prevotella copri* is prevalent in the oral cavity and intestines of RA patients [4,5], whereas others have shown that *Prevotella histicola* may reduce the risk of developing RA in mice [6]. This, in turn, shows that different types of bacteria belonging to the same genus may have different effects on autoimmunity in RA. Chen et al. also found that RA and osteoarthritis (OA) patients had an increased presence of *Prevotella* and *Leptotrichia* taxa compared to healthy controls [7]. In a Swedish patient group, an increase in the relative abundance of *Capnocytophaga leadbetteri*, *Prevotella dentalis*, *Prevotella intermedia*, *Prevotella melaninogenica*, *Parvimonas micra, Fusobacterium subsp. vincentii*, and the *Saccharibacteria* (TM7) [G-1] bacterium HMT349 was observed in the RA group [5]. In contrast, Sharma et al. reported that *Leptotrichia* was significantly depleted in patients with Sjögren’s syndrome compared to the control group [8], whereas *Veillonella* was elevated in patients with Sjögren’s syndrome according to a study by Singh et al. [9]. It should be noted that the presence of Sjögren’s syndrome worsens the course and prognosis of RA.

Peng et al., analyzing and systematizing the available research data, concluded that the microbial composition of the oral cavity of patients with RA is influenced by the disease [10]. The study suggests that there may be a link between the oral microflora and RA. [11]. This is supported by previous research showing a lower diversity of microbial species in the oral cavity of patients with RA compared to healthy individuals [2]. However, other studies have detected heat shock proteins (e.g., hsp 70) of certain oral bacteria, such as *Prevotella nigrescens* and *Prevotella intermedi,* in the blood serum and the synovial fluid [12]. In addition, DNA of *P. gingivalis, Tannerella forsythia* and *P. intermedia* was found in samples of synovial fluid in patients with RA [13]. This suggests that bacteria from the oral cavity may be able to migrate into the articular tissues. In this paper, we report a pilot case–sectional study comparing the diversity and composition of the oral microbiome in local patients diagnosed with RA to that of healthy individuals. This study, to our knowledge, is the first of its kind to be conducted in Kazakhstan and Central Asia.

## 2. Materials and Methods

### 2.1. Ethics Statement

The study was approved by the local ethics committee of the National Laboratory Astana Nazarbayev University, Protocol No. 03-2019, Astana, Kazakhstan. Respondents were informed about the aims of the study and signed an informed consent form. Interaction with respondents was carried out in accordance with the general instructions and rules.

### 2.2. Participants and Recruitment

**Figure 1:**
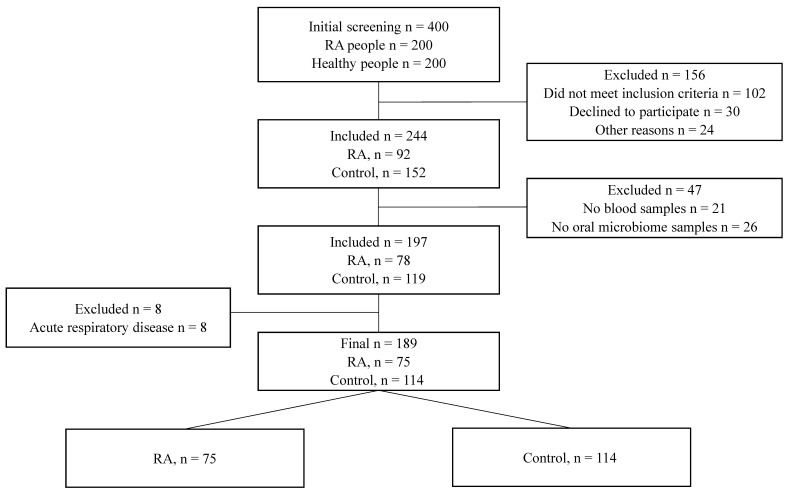
STARD diagram showing the flow of participants through the study.

Patient recruitment was conducted from outpatients No. 10 and No. 12 of Astana city, Kazakhstan, and via an official invitation on social networks in the period from September 2019 to December 2021. Overall, 189 individuals, including 75 RA patients and 114 healthy controls, were included in the study (Figure 1). Considering the inclusion criteria (established RA diagnosis with a duration of more than 1 year) and exclusion criteria (pregnancy, lactation, oncology, comorbid conditions, decompensated chronic diseases, taking antibiotics and probiotics 3 months before the study), healthy controls lacked heredity for autoimmune diseases, including RA. Anamnestic and examination data were recorded in the individual cards with the assignment of an individual code. Examination of the bone and joint system was carried out according to generally accepted rules. The tender joint number (TJC) and swollen joint number (SJC) were counted. The symptoms of transverse compression of the hands and feet and the strength of compression of the hands were evaluated. The deformities and disfigurations were recorded. Data on extra-articular manifestations and the presence of complications were recorded. The pain assessment was carried out using a visual analog scale, and the overall average score was registered. The assessment of disease activity was carried out according to the disease activity score (DAS28) disease activity index. Active RA was defined as DAS28 ≥2.6, and RA disease in remission was defined as DAS28 <2.6 [14]. The X-ray stage was set according to the X-ray images of patients over the past year.

All patients underwent laboratory examination. Blood sampling was carried out strictly in the fasting state after a 12–14 h period of fasting. The inflammatory marker C-reactive protein (CRP) was examined. Immunological parameters such as RF and ACPA were also determined. The diagnosis of RA in the patients under the study was established according to 2010 rheumatoid arthritis classification criteria [15].

### 2.3. Sample Processing and Sequencing

Prior to sample collection, respondents were advised not to perform oral hygiene during the last 12 h and not to eat or drink during the last 2 h prior to the study visit. During the visit, scrapings were collected from the surface of the tongue, gums, tonsils, and plaque. Oral scraping samples were collected in a DNA/RNA Shield Collection Tube w/Swab (Zymo Research, R1107). Genomic DNA from oral samples was extracted using the ZymoBIOMICS DNA Miniprep Kit (Zymo Research, D4300). A qualitative control of DNA isolation was performed by electrophoresis in a 1% agarose gel. The concentration and purity of each DNA sample were determined using an Invitrogen Qubit 3.0 Fluorometer (Invitrogen, Carlsbad, CA, USA). Sterile water served as a negative control. Sequencing was performed on the Illumina NovaSeq 6000 platform at the laboratory of Novogene (Beijing, China) following the standard Illumina protocols.

### 2.4. Assessments of the Periodontal Status in the RA Patients and Control Cohorts

Clinical examination of the participants was conducted by a dentist, and a thorough collection of complaints and anamnesis of the disease, an examination of the oral cavity, and an index assessment of the condition of the tissues of the periodontal complex were carried out. The survey considered patients’ complaints about bleeding gums, the prescription and conditions of its occurrence, the frequency of abscess formation, the presence of bad breath, and the mobility of teeth, as well as a cosmetic defect associated with the movement of the frontal group of teeth, increased sensitivity of teeth from all types of irritants, and a violation of the function of the dental system. To clarify the diagnosis, if necessary, an X-ray examination was performed. Being periodontally healthy was defined as areas with a probing depth of ≤3 mm and the absence of bleeding during probing [16]. The presence of periodontitis was assessed in cases of an areas with a depth of probing ≥4 mm and a clinical loss of attachment ≥2 mm and radiographic bone loss < 15%.

### 2.5. Processing of Sequencing Data

The LotuS2 pipeline (Less OTU Scripts 2) [17] was used to process 16S amplicon sequencing data from raw reads into taxon density tables. Demultiplexing, quality filtering, and dereplication of reads were implemented using a simple demultiplexer (sdm). Chimeras were removed using algorithms for detecting chimeric sequences UCHIME. Taxonomic postprocessing of amplicon sequences in LCA with sequence clustering UPARSE was performed using the SILVA database.

Alpha diversity (the richness of a sample in terms of the diversity of OTUs observed in it) was estimated using Shannon and Simpson indices. Beta diversity was measured using weighted UniFrac [18] on Hellinger transformed data [19]. Principal coordinates analysis (PCoA) with weighted UniFrac distance was used to examine the separation of operational taxonomic units across samples. The association between microbiome composition and the study group was tested using PERMANOVA with 9999 permutations. Differences in the relative abundance of the microbial features were determined by linear discriminant analysis (LDA) effect size (LEfSe). The threshold on the logarithmic LDA score for distinguishing features was set to 2.0 [20].

### 2.6. Functional Capabilities of the Oral Microbiome

Functional metagenomes were predicted based on the 16S rRNA sequencing data of the oral microbiome using PICRUSt2 (phylogenetic investigation of communities by reconstruction of unobserved states) v2.5.0 with default parameters [21]. Briefly, the AVSs were placed into a reference tree (NSTI cutoff value of 2) containing 20,000 full 16S rRNA sequences from prokaryotic genomes, which was then used to predict individual gene family copy numbers for each AVS. The predictions are based on the Kyoto Encyclopedia of Genes and Genomes (KEGG) orthologs (KO). The produced KEGG orthologs (KOs) were mapped to the KEGG module annotation downloaded on 1 April 2022 from the KEGG BRITE database [22].

### 2.7. Statistical Analysis 

R v4.2.0 (R Foundation for Statistical Computing, Vienna, Austria) was used for statistical evaluation. For the correlation between bacterial abundance and blood/serological indicators, the Spearman correlation coefficient was used. Continuous data were compared using unpaired *t*-tests or Mann–Whitney U tests, as appropriate, whereas chi-squared tests or Fisher’s exact tests were used to compare discrete parameters. All results were considered statistically significant when *p*-values were less than 0.05.

## 3. Results

### 3.1. Subjects of the Study, Characteristics

The observational study included 189 participants, with 75 diagnosed with rheumatoid arthritis and 114 being healthy individuals (as shown in Figure 1). The average age of RA patients was 46 years, with the onset of the disease typically occurring in the third decade of life. One third of the RA patients had a family history of the disease. The majority of the RA patients (81.3%) had moderate-to-high disease activity. Despite previous studies linking periodontitis with RA development, our sample did not show a significant association. Accordingly, only 21.3% of RA patients and 17.5% of healthy controls had been diagnosed with periodontitis [23,24]. The patients were primarily treated with methotrexate, methylprednisolone, and nonsteroidal anti-inflammatory drugs, in accordance with the clinical protocol for RA diagnosis and treatment in the Republic of Kazakhstan. [25].

The control group consisted of individuals who were matched for age, sex, and ethnicity to the RA patients, but did not have a history of autoimmune diseases or the use of antibacterial drugs or probiotics in the three months prior to this study (Table 1). 

They also did not have diabetes mellitus or oral diseases. Out of the 75 RA patients in the study cohort, 76% tested positive for RF and 24% tested negative. The results also showed that 57.3% of patients were ACPA-positive, whereas the remaining 42.7% of RA patients had ACPA-negative forms of the disease (Table 1). 

### 3.2. Microbial Profile of the Oral Cavity in Rheumatoid Arthritis

A total of 15,616,320 taxonomic marker tags were analyzed, witch 14,517,729 sequences remaining after sequence denoising, trimming, and chimera filtration. The average sequencing depth was 75,208 reads per sample (IQR = 48 168–103 578). Using distance similarity ≥97%, the sequences were grouped into 2049 OTUs, of which 2042 OTUs belonged to the bacterial kingdom.

**Figure 2:**
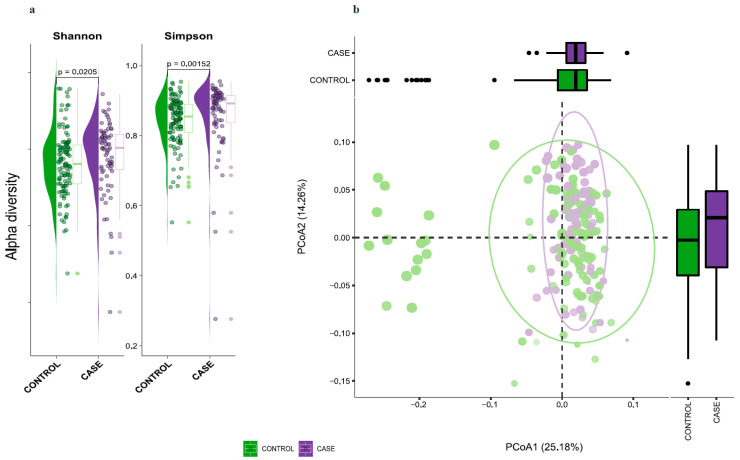
Microbial biodiversity of the oral cavity. (**a**) The 𝛼-diversity distribution among incident RA patients and healthy controls (case); (**b**) β-diversity of the oral microbiome in RA patients and cases. The points with green illumination belong to the samples of the control group, and the purple points belong to the RA group.

Comparison of the oral microbiota of the two groups (healthy controls and RA patients) detected 473 taxa at 7 taxonomic ranks. Bacterial richness (𝛼-diversity), estimated using the Shannon and Simpson biodiversity indices, showed a significant difference between the two groups (*p* < 0.02 and *p* < 0.001, respectively) (Figure 2a). Interestingly, taxonomic biodiversity was found to be statistically higher in the RA group compared to the healthy group. Comparisons of the similarity of the two communities at the group level showed a α-diversity *p* < 0.05 and α-similarity (dispersion) *p* > 0.05 (Figure 2b).

**Figure 3:**
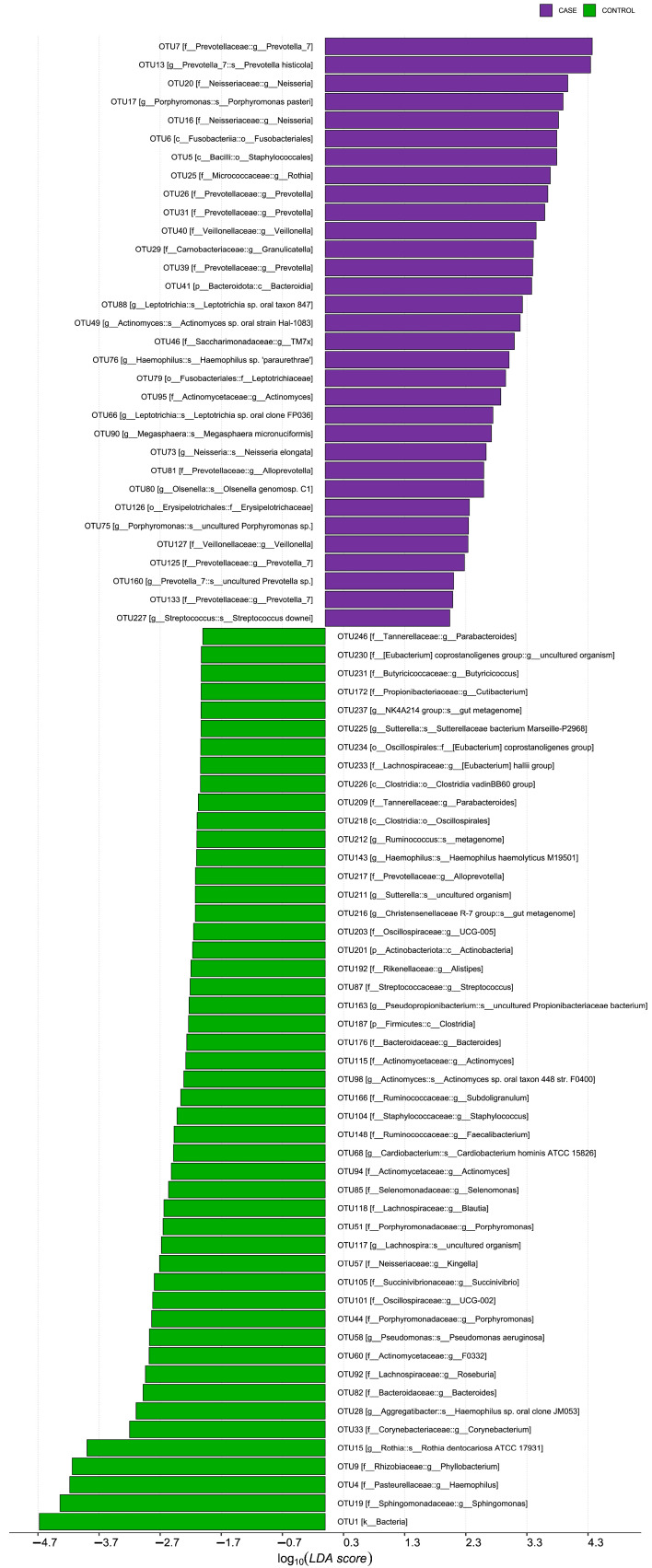
Taxonomic profile of differences in patients with rheumatoid arthritis. A histogram of LDA scores, where the LDA score indicates the size and ranking of each differentially abundant taxon (LDA > 2). The OTU case group (RA) is highlighted in green and the control group (non-RA) is highlighted in purple.

Since the bacterial diversity (𝛼-diversity) differed between the groups, we carried out the determination of statistically altered bacterial taxa using LEfSe [20]. A histogram representing the structure of the oral microbiota is shown in Figure 3. The LDA score histogram was calculated to determine any signs of differential abundances between RA patients and the healthy group. Significant differences in taxa at different taxonomic levels between the two groups are shown. The oral microbiome of RA patients was characterized by an increase in the relative abundance of *Prevotellaceae* and *Leptotrichiaceae*, as well as the persistence of *Neisseria*, *Fusobacteriales*, *Rothia*, *Granulicatella*, *Leptotrichia*, *Megasphaera micronuciformis*, *Olsenella*, *and Erysipelotrichaceae*. Conversely, several butyrate- and propionate-producing taxa, including *Eubacterium coprostanoligenes*, *Eubacterium halli*, *Butyricicoccus*, *Cutibacterium*, *Ruminococcus*, *Subdoligranulum*, *Faecalibacterium*, *Blautia*, *and Roseburia*, were depleted in RA patients, but enriched in healthy controls.

### 3.3. Relationship between Oral Microbiota and Disease Activity, CRP, Autoantibodies

Next, we investigated the relationship between the microbial composition of the oral cavity and the disease activity score of patients with RA. We found that certain microbial taxa were associated with different stages of disease activity. For example, RA patients with low disease activity (DAS28 score < 2.6; n = 8) had an increase in *Treponema* sp. (*p* = 0.01527) and *Absconditabacteriales* (SR1) (*p* = 0.03510). Those with moderate disease activity (DAS28 score 2.6–3.2; n = 6) had an increase in *Porphyromonas* (*p* = 0.03213), and those with high disease activity (DAS28 score > 5.1; n = 26) had an increase in *Staphylococcus* (*p* = 0.04127), whereas the moderate stage did not differ in the statistically increased content of any taxa in the studied sample (Supplement Table S1). 

**Figure 4:**
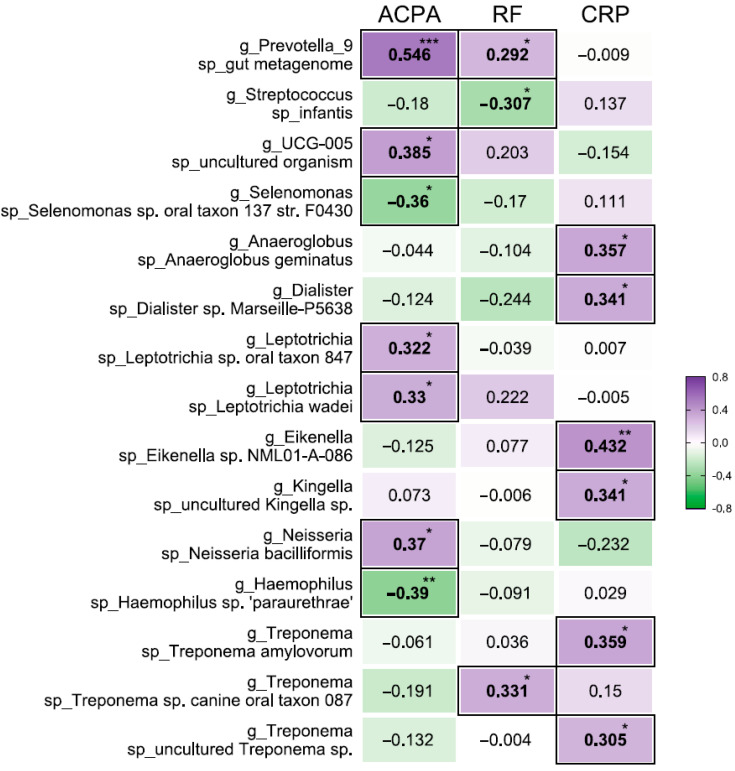
Association between oral bacterial abundance and systemic signature in patients with RA. Correlation between the relative abundance of certain taxa and concentrations of ACPA and RF and C-reactive protein (CRP) in serum. The color scale represents the magnitude of the correlation. Purple highlights indicate a positive correlation; green highlights indicate a negative correlation. * *p* < 0.05, ** *p* < 0.01, *** *p* < 0.001.

In addition, we analyzed the relationship between microbial toxins and the presence of autoantibodies (ACPA and RF) and inflammation (measured by CRP) in the blood of these patients. The presence of these autoantibodies is thought to be associated with RA disease severity, and accumulating evidence links certain oral microorganisms to the generation of ACPA and RF [26]. No correlations were observed at the OTU level, but 15 taxa at the species level were found to be associated with at least one of the parameters (Figure 4). 

For example, *Prevotella_9* was positively correlated in a statistically significant manner with high levels of ACPA and RF in the blood (*p* < 0.001). *Oscillospiraceae* UCG-005, *Leptotrichia* sp. oral taxon 847, *Leptotrichia wadei*, and *Neisseria bacilliformis* also had moderate positive correlations with high ACPA levels. On the other hand, *Selenomonas* sp. and *Haemophilus* sp. ‘paraurethrae’ were moderately negatively correlated with ACPA levels. A weak positive correlation was further observed between RF and *Prevotella_9*, and a moderate positive correlation was observed between *Treponema* sp. canine oral taxon 087 and RF. *Streptococcus infantis*, on the other hand, had a moderate negative correlation with RF. It is notable that *Anaeroglobus geminatus*, taxon family N*eisseriaceae Dialister* sp. Marseille-P5638, *Eikenella* sp. NML01-A-086), taxon family *Spirochaetaceae*: *Treponema amylovorum*, uncultured *Treponema* sp. all had a moderate positive correlation with CRP. 

### 3.4. Functional Profiles of the Oral Microbiome Associated with RA

**Figure 5:**
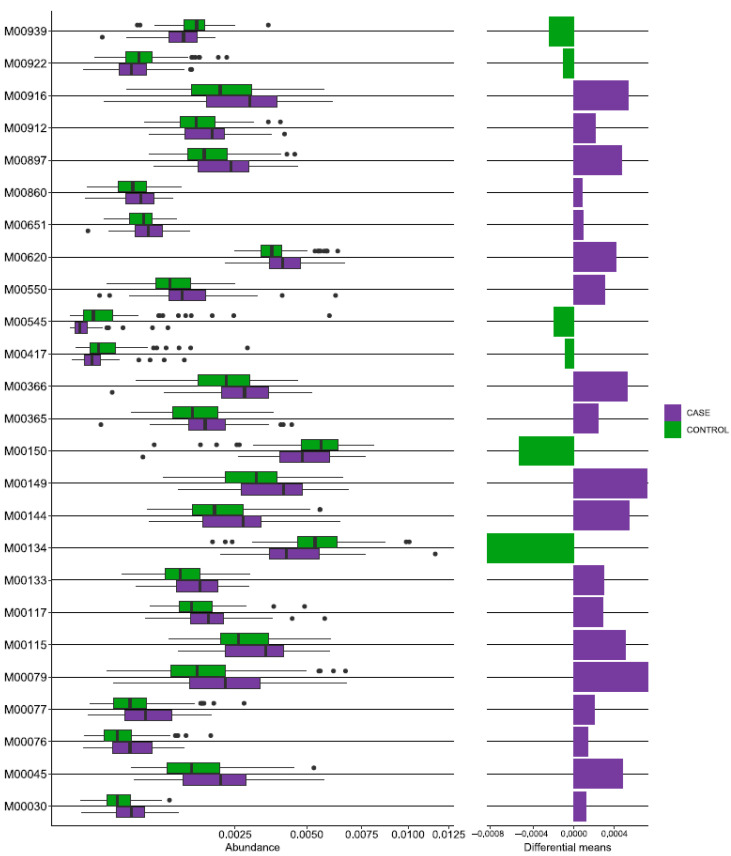
Functional repertoire prediction of differential KEGG levels by 16S sequencing in the rheumatoid arthritis group. The boxplot shows a comparison of PICRUSt2 predicted KEGG functions between the case (RA) and control groups. The case group is highlighted in purple, and the control group in green.

In order to investigate the functional role of the oral microbiome in RA, we used the PICRUSt2 tool to perform functional prediction based on marker sequences of the 16S rRNA gene [21]. This analysis identified 6119 KEGG orthologs (KOs) (Supplemental Table S2) and 457 modules (Supplemental Table S3), which were then filtered to identify truly different KEGG modules (fraction of module at least 0.1% of total number of modules, difference between modules at least 10%, FDR = 0.1). As a result, we found that 25 KG modules were significantly different in the RA group compared to the healthy control group, with 19 being increased and 6 being decreased (Figure 5).

The functional pattern of patients with RA showed increased activity in the biosynthesis of vitamins (B6, precursor B3, B1, ubiquinol, quinolinate), carbon fixation, carbohydrate metabolism (ascorbate degradation), glycan metabolism (keratan, dermatan, chondroitin sulfate degradation), histidine, and lysine metabolism. However, there was decreased activity in pyrimidine metabolism, xenobiotic biodegradation (aromatic degradation), energy metabolism, and overall amino acid metabolism. 

RA studies have shown that high levels of ACPA and RF in the blood of patients are associated with a more severe and aggressive form of the disease, as well as reduced remission [27]. Patients who are only positive for ACPA or only positive for RF tend to have an intermediate course of the disease [28]. To examine whether there were differences in the functional profile of individuals who tested positive for these antibodies, the study group was divided into four subgroups based on their ACPA and RF status: ACPA−/RF− (n = 15), ACPA−/RF+ (n = 17), ACPA+/RF− (n = 3), ACPA+/RF+ (n = 40), and control (n = 114).

**Figure 6:**
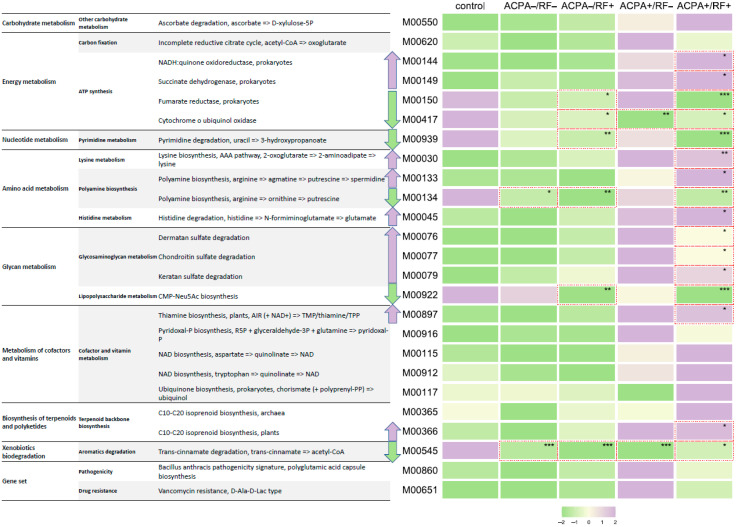
Differential patterns predicted by KEGG modules in combinations of anticitrullinated protein antibodies and rheumatoid factor. The abundance of predicted functional modules is shown on the basis of color illumination, where green indicates a decrease in the function parameter, and purple indicates an increase. Trends toward an increase or decrease in the abundance of predicted functions are shown by highlighted arrows. * *p* < 0.05, ** *p* < 0.01, *** *p* < 0.001. The ACPA+/RF− group includes only three samples, so statistical indicators can be both representative and distort the picture associated with characteristic changes.

Interestingly, the ACPA+/RF− and ACPA+/RF+ groups had a higher predicted presence of genes involved in ascorbic acid degradation, energy metabolism such as succinate dehydrogenase and fumarate reductase, and the tricarboxylic acid cycle and respiratory electron transport chain. There was also an increase in the module for lysine and polyamine biosynthesis. The presence of taxa that degrade glycosaminoglycans, such as dermatan, chondroitin, and keratan, was significantly increased and widely distributed in tissues, particularly in the bones and cartilage of vertebrates. These molecules contribute to the gelation of the extracellular matrix in these tissues [29], which may suggest that the microbiota plays a role in the development and pathogenesis of RA in seropositive patients. Taxa producing thiamine pyrophosphate, pyridoxal-p, NAD, and ubiquinol were also significantly increased. In contrast, the ACPA−/RF− and ACPA−/RF+ groups had a reduced functional repertoire compared to both the healthy group and the ACPA+/RF− and ACPA+/RF+ groups (Figure 6, Supplemental Table S3).

## 4. Discussion

In this study, the oral microbiome of Kazakhstani patients with RA was analyzed using 16S rRNA gene sequencing. The results showed an increased presence of *Prevotellaceae*, *Leptotrichiaceae* and *Neisseria*, *Fusobacteriales*, *Rothia*, *Granulicatella*, *Leptotrichia*, *Megasphaera micronuciformis*, *Olsenella*, *and Erysipelotrichaceae* in the oral microbiome of these patients. This is in line with previous research indicating that the oral microbiome plays a role in the development or progression of RA, possibly through dysbiosis of the oral cavity and concomitant periodontal disease [30]. Interestingly, the abundance of *Porphyromonas*, particularly *Porphyromonas gingivalis*, was low in the study group despite the bacterium being linked to systemic autoimmune diseases and the potential to contribute to the development of autoimmunity and clinical RA [31]. 

Previous research has shown that the *Prevotellaceae* taxa in RA show a feedback relationship with *Bacteroides* [32], which may explain the small presence of *P. gingivalis* in our RA cohort. Additionally, Kishikawa et al. and other authors have found a higher relative abundance of the *Prevotella* taxon in the RA group [32,33]. A higher relative abundance of *Prevotella*, *Veillonella*, and *Porphyromonas* was also reported in samples of patients with early RA and individuals at risk compared with a healthy control group [3]. 

In our study, we observed significant dissimilarity in the microbiota of healthy individuals and RA patients, with higher biodiversity in the RA group. The RA group also had an increase in the average diversity of taxa according to the Simpson index (*p* = 0.00152) and Shannon index (*p* = 0.0205) compared to the control group, which may be linked to dysbiosis of the oral cavity and potentially periodontal disease (although periodontal disease was not specifically studied). These opportunistic microorganisms are known to cause periodontal and dental issues [34]. Whereas group similarity, as measured by the α-similarity index, did not show significant differences, group diversity, as measured by the α-diversity index, did show statistically significant differences. 

In this study, a relationship was found between the activity of disease and certain taxa of bacteria. Specifically, *Treponema* sp. (*p* = 0.01527), *Absconditabacteriales* (SR1) (*p* = 0.03510), *Porphyromonas* (*p* = 0.03213), *Staphylococcus* (*p* = 0.04127), *Anaeroglobus geminatus*, *Prevotella/Leptotrichia*, *Oscillospiraceae* UCG-005, and *Neisseria bacilliformis* (*p* = 0.05) were all correlated with the activity of the disease. Picchianti Diamanti et al. have previously shown that the relative abundance of intestinal microbial taxa was associated with the DAS28 score [35]. Previous research has also shown that some of these taxa, such as *Porphyromonas* and *Treponema* sp., have been previously associated with the active stage of the disease [36] and are known to induce periodontal diseases and the production of citrullinated antigens. Representatives of these taxa, according to the literature data, have solidary (neuraminidase) properties and can induce periodontal diseases and the production of citrullinated antigens. For instance, *P. gingivalis* is able to aggravate the proinflammatory reaction of the body through the activation of a Toll-like receptor (TLR)2/4 [37]. Another report also noted that the detection of higher proportions of *Treponema* sp. taxa in patients with DAS28 scores <2.6 is likely associated with new onset of RA [38]. In addition, the relative abundance of *Streptococcus infantis* in the oral microbiota was found to be negatively correlated with the concentration of a certain factor in the blood of patients with arthritis. Overall, these findings suggest that certain bacteria may be involved in the development and progression of the disease.

Brusca et al., in their review [39], drawing conclusions based on previous studies, reported that the presence of taxa such as *Anaerglobus geminatus* and *Prevotella/Leptotrichia* has been linked to ACPA seropositivity and clinical RA. Our own research also found correlations between *Prevotella/Leptotrichia* with the level of clinical RA and ACPA positivity, as well as between taxa such as *Oscillospiraceae* UCG-005 and *Neisseria bacilliformis*. Tong et al. observed an increase in *Prevotella* abundance in Chinese patients associated with RA progression [2]. Our study further highlights the association of *Prevotella* with the level of RF in blood serum. However, the relative abundance of *Streptococcus infantis* in the oral microbiota was negatively correlated (*p* < 0.01) with the concentration of RF in patients with arthritis.

Prediction of the potential functional profile of the oral microbiota showed a change in some functions in the RA cohort. These changes included a reduction in the metabolism of pyrimidine, the biosynthesis of polyamines and polysaccharides, and the biodegradation of xenobiotics. In addition, the study found an increase in the level of vancomycin resistance genes and virulence factors. Further analysis of the oral microbiota in RA patients revealed differences between subgroups based on the presence of ACPA and RF autoantibodies. The ACPA+/RF− and ACPA+/RF+ subgroups had an increased presence of bacteria that metabolize glycosaminoglycans (dermatan, chondroitin, keratan), which are important components of joint tissue. This suggests that the oral microbiota may contribute to inflammation and joint tissue damage in RA through the transmission of bacterial taxa between the mucous membranes of the oral cavity and the gut [40], as well as through increased intestinal permeability in the late stages of RA. However, the reduced presence of certain bacteria in other subgroups suggests that the role of the oral microbiota in RA may be complex and requires further analysis.

In this study, we also observed a reduced presence of the lipopolysaccharide metabolism module in certain subgroups of patients with long-standing RA. Our findings are consistent with previous research [41,42] showing that the microbiota of these patients have an increased ability to degrade ascorbate, which may contribute to disease development. According to the study by Pattison et al., it was reported that ascorbate prevents the development of inflammatory arthritis [43]. It is important to note that patients with rheumatoid arthritis typically experience a deficiency of ascorbate and require high doses of supplements to maintain an adequate level of ascorbate in the plasma.

There are several limitations to this study. One major limitation is that all of the patients in the study had a long course of RA, which tends to be associated with a less aggressive disease course. Additionally, the patients were receiving treatment at the time of the study (including nonbiologic DMARDs such as methotrexate and leflunomide, biologic DMARDs such as infliximab, and symptomatic therapy such as NSAIDs and GCS), whereas the control group of volunteers did not receive any treatment. In addition, the ACPA+/RF− group included only three patients, which is a small sample size. Finally, the functional repertoire of the microbiome was only evaluated based on 16S rRNA sequence profiles, without determining the functional abundance of the corresponding genes in the microbial community.

## 5. Conclusions

Our study found that patients with RA have a different composition of microbes in their oral cavity compared to non-arthritic individuals. Specifically, the microbial signature in the study cohort was characterized by a decrease in the number of butyrate- and propionate-producing microorganisms, but an increase in overall 𝛼-diversity. The microflora was further distinguished by the enrichment of taxa such as *Prevotellaceae* and *Leptotrichiaceae*. This, in turn, once again, confirms the role of the *Prevotella* taxon in the development of RA. In addition, an association of the microbial signature with disease activity and ACPA and RF autoantibody profiles was found. These findings suggest that it is important to consider the profile of a patient’s oral microbiome when choosing a treatment strategy for RA, in order to provide a personalized approach.

## Figures and Tables

**Table 1:** Demographic and clinical characteristics of the studied groups.

Parameters	RA Patients (*n* = 75)	Control (*n* = 114)	*p*-Value
Demographic characteristics			
Mean age (years)	46 (Q1–Q3: 38–50)	43 (Q1–Q3: 37–49)	0.473
Age of RA onset	34 (Q1–Q3: 26–43)	-	
Disease duration (years)	6 (Q1–Q3: 3–12)	-	
Smokers (%)	4 (6)		
Heredity for RA	26 (34.7%)		
BMI (kg/mkv)	24.5 (Q1–Q3: 21.6–28.4)	25.5 (Q1–Q3: 22.6–28.4)	0.351
Periodontitis (PD)	16 (21.3%)	20 (17.5%)	0.516
Periodontally healthy (PH)	59 (78.7%)	94 (82.5%)	
**Inflammatory marker**			
CRP (mg/L)	3(Q1–Q3:0.75–7.65)	1(Q1–Q3:0.4–2)	<0.001
**Autoantibodies**			
RF positive (%)	57 (76.0%)	-	
ACPA positive (%)	43 (57.3%)	-	
**Disease activity**		-	
DAS28 score > 5.1	28 (37.3%)	-	
DAS28 score 3.2–5.1	33 (44.0%)	-	
DAS28 score 2.6–3.2	6 (8.0%)	-	
DAS28 score < 2.6	8 (10.7%)	-	

## Data Availability

The 16S rRNA amplicon sequencing data from this study have been deposited in the NCBI BioProject under accession number PRJNA841771.

## Supplementary Materials

The following supporting information can be downloaded at: https://www.mdpi.com/article/10.3390/jcm12113694/s1, Table S1: RA disease activity score (DAS28) and oral microbial signature.
