# Supplementary figures and images for: Oral Microbial Signature of Rheumatoid Arthritis in Female Patients
Source: J Clin Med. 2023 May 26;12(11):3694. doi: 10.3390/jcm12113694 (PMC10253734; doi:10.3390/jcm12113694)

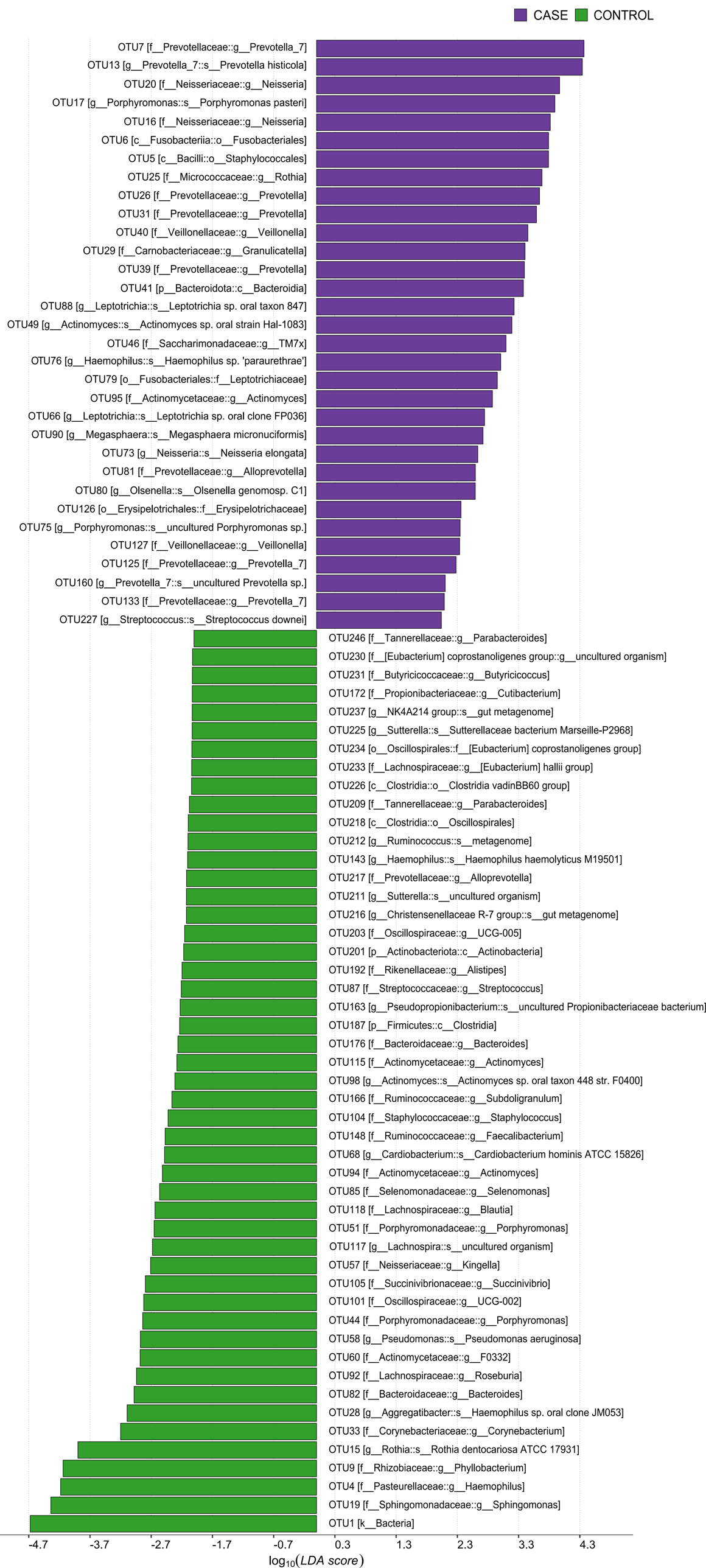

Supplement: Supplementary file 1 [file jcm-12-03694-s001.zip › jcm-2331802-supplementary.pdf]
